# The representation of time windows in primate auditory cortex

**DOI:** 10.1093/cercor/bhab434

**Published:** 2021-12-07

**Authors:** Pradeep Dheerendra, Simon Baumann, Olivier Joly, Fabien Balezeau, Christopher I Petkov, Alexander Thiele, Timothy D Griffiths

**Affiliations:** Biosciences Institute, Newcastle University, Newcastle upon Tyne, NE2 4HH, UK; Institute of Neuroscience and Psychology, University of Glasgow, Glasgow G128QB, UK; National Institute of Mental Health, NIH, Bethesda, MD 20892-1148, USA; Department of Psychology, University of Turin, Torino 10124, Italy; Biosciences Institute, Newcastle University, Newcastle upon Tyne, NE2 4HH, UK; Biosciences Institute, Newcastle University, Newcastle upon Tyne, NE2 4HH, UK; Biosciences Institute, Newcastle University, Newcastle upon Tyne, NE2 4HH, UK; Biosciences Institute, Newcastle University, Newcastle upon Tyne, NE2 4HH, UK; Biosciences Institute, Newcastle University, Newcastle upon Tyne, NE2 4HH, UK

**Keywords:** functional magnetic resonance imaging (fMRI), primates, time-window processing

## Abstract

Whether human and nonhuman primates process the temporal dimension of sound similarly remains an open question. We examined the brain basis for the processing of acoustic time windows in rhesus macaques using stimuli simulating the spectrotemporal complexity of vocalizations. We conducted functional magnetic resonance imaging in awake macaques to identify the functional anatomy of response patterns to different time windows. We then contrasted it against the responses to identical stimuli used previously in humans. Despite a similar overall pattern, ranging from the processing of shorter time windows in core areas to longer time windows in lateral belt and parabelt areas, monkeys exhibited lower sensitivity to longer time windows than humans. This difference in neuronal sensitivity might be explained by a specialization of the human brain for processing longer time windows in speech.

## Introduction

Primate vocalizations contain features that vary over time at different rates. The ability to extract, represent, and recognize acoustic features depends on the time windows used for analysis of the acoustic signal. Short time windows provide higher temporal resolution for the analysis of rapidly changing features and enable quicker responses, while long time windows provide higher resolution of spectral features and better signal-to-noise ratio for slowly changing acoustic features. The optimal duration of a time window therefore depends upon the underlying acoustic features that need to be processed. Here, the window duration is operationalized as time required for the correlation between amplitude spectra to recede to a target value (see Materials and Methods). Both human and monkey calls contain features at a range of different rates. However, the prominence of different rates differs between the two species. We consider here whether human and nonhuman primates share a common functional anatomy to support the analysis of different-length time windows and whether this anatomy is adapted in the two species to reflect differences in the time windows needed to process species-specific vocalizations.

Consideration of the structure of vocalizations provides clues to the time windows that might be emphasized in different primate species. For humans, speech contains prominent low frequency modulations in the range of 2–8 Hz which are relevant to the syllabic rates of speech that humans produce (Rosen 1992; Chandrasekaran et al. 2009; Elliott and Theunissen 2009; Ding et al. 2017; Poeppel and Assaneo 2020). A recent theory posits a neural oscillator in speech motor cortex at a syllabic rate which feeds back to auditory cortex in a way that might emphasize perceptual analysis at this slow rate (Poeppel and Assaneo 2020). In humans, Elliott and Theunissen (2009) reported temporal modulations in speech between 1 and 7 Hz are most important for intelligibility. For macaques, Cohen et al. (2007) reported that the between-call variance in macaque vocalization classes was high at temporal modulation frequencies between 5 and 20 Hz, suggesting that macaque conspecific vocalizations (sounds produced by the same species) depend less on low temporal modulation rates. Joly et al. (2012) reported that behaviorally relevant macaque vocalizations can be very dissimilar to human speech because they contain faster temporal modulations. These observations suggest that the analysis of longer time windows is emphasized in the analysis of communication sounds in humans compared with macaques.

**Figure 1:**
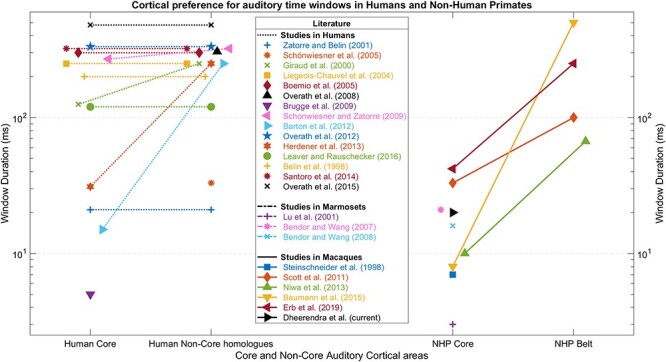
Visual summary of cortical preference for auditory time windows in humans and nonhuman primates. The studies employ a variety of stimulus types to infer the preference of auditory cortical areas for different time-window analysis. The demonstration of preferred time windows is not always possible. Compared with nonhuman primate studies, most studies in humans suggested a preference for longer windows. However, within both species, the core tended to prefer shorter windows even though this difference was more consistently found in nonhuman primates. Studies in humans: Zatorre and Belin (2001)—narrowband tones; Jamison et al. (2006)—narrowband tones, Schönwiesner et al. (2005)—narrowband tones, Giraud et al. (2000)—AM broadband noise, Liegeois-Chauvel et al. (2004)—AM broadband noise, Boemio et al. (2005)—broadband noise, Overath et al. (2008)—broadband tones, Brugge et al. (2009)—click trains, Schonwiesner and Zatorre (2009)—dynamic ripples, Barton et al. (2012)—AM broadband noise, Overath et al. (2012)—AM broadband noise, Herdener et al. (2013)—AM broadband noise, Leaver and Rauschecker (2016)—AM broadband noise, Belin et al. (1998)—pseudo speech, Santoro et al. (2014)—natural sounds and vocalizations, and Overath et al. (2015)—speech and natural sounds. Studies in marmosets: Lu et al. (2001)—click trains, Bendor and Wang (2007)—pulse trains, and Bendor and Wang (2008)—narrowband AM tones. Studies in macaques: Steinschneider et al. (1998)—click trains, Scott et al. (2011)—AM narrowband tones, Niwa et al. (2013)—AM broadband noise, Baumann et al. (2015)—AM broadband noise, and Erb et al. (2019)—natural sounds and vocalizations. Descriptions of each study are presented in the supplementary text.

Natural communication sounds have been used in brain experiments in humans and macaques. Figure 1 shows studies relevant to time-window analysis in auditory cortex (details about each study included in this literature survey are in the supplementary text), including studies based on conspecific sounds. There are disadvantages in using such sounds, particularly in work designed to investigate species differences in time-window processing. They might engage top-down mechanisms that are species-specific, and control over spectrotemporal properties is not possible in the same way as in synthetic stimuli (but, see Nourski et al. (2009)).

Studies using AM sounds allow a comparison between species using temporally varying stimuli without species-specific significance. This allows comparison of an extensive human psychophysical literature from the 1950s (Zwicker 1952) and more recent behavioral work in primates. O'Connor et al. (2011) examined the detection of sinusoidal AM of broadband noise in macaques and demonstrated lower sensitivity to low modulation rates and greater sensitivity to high modulation rates in macaques compared with humans.

A number of the brain studies of temporal analysis in human and macaques shown in Figure 1 have used modulated sounds. Many of the macaque studies suggest an increase in optimal time window from posterior core to anterior belt areas, but the human studies using modulated stimuli do not show such a consistent pattern of differences between posterior core and anterior belt homologs.

In this experiment, we used a broadband “spectral flux” stimulus containing fluctuations in the spectrum over time, which is more like natural sounds than deterministic modulations but without any semantic confound. This allows estimates of the time window to which areas of the brain are most sensitive using a stimulus suitable for any species. In humans, Overath et al. (2008) reported a lack of differential sensitivity to time windows in human core homologs and sensitivity to longer time windows in belt and parabelt homologs in planum temporale, anterior superior temporal gyrus, and right superior temporal sulcus (STS). This supports a specialization for longer time windows in homologs of noncore auditory cortex.

We used the spectral flux stimulus to investigate time-window analysis in macaques using functional magnetic resonance imaging (fMRI). The data show systematic changes in tuning to different time windows of analysis between core and noncore cortex in the macaque. But the pattern of change is different from humans: Humans show no preference in core homologs and a preference for long time windows in noncore homologs, while macaques show a preference for short time windows in core areas and no preference in parabelt areas. We suggest a model based on a common gradient of preferred time windows across the auditory cortex of primates, which has been adapted in humans to support the analysis of long windows required for speech in high-level auditory cortex.

## Materials and Methods

All procedures conducted with the macaques were approved by the Animal Welfare and Ethical Review Body at Newcastle University and the UK Home Office and are in full compliance with both the UK Animal Scientific Procedures Act and the European Directive (2010/63/EU) on the care and use of animals in research. We support the principles of the consortium on Animal Research Reporting of In Vivo Experiments.

Given the ethical sensitivities involved in research with nonhuman primates and the 3Rs principles (one of which is on the Reduction of animal numbers), our work with awake behaving macaques requires using the fewest macaques necessary. A sample size of two to three is common in behavioral neuroscience experiments with macaques, provided that results are robust within each individual and that the effects generalize beyond one animal, as they do. Training macaques for awake magnetic resonance imaging (MRI) scanning requires a substantial time investment, and the data that were combined for each of these datasets in each of the three animals are statistically robust and consistent across the three animals. Thus, there was no ethical justification to train and test additional monkeys.

### Subjects

The imaging data were obtained from scanning sessions with three male rhesus macaques (*Macaca mulatta*) denoted as M1 (12-year-old male weighing 17 kg), M2 (9-year-old male weighing 16 kg), and M3 (9-year-old male weighing 10 kg). The animals have been previously habituated to the scanner environment as well as exposed to some experimental auditory stimuli prior to scanning. Further, they had been trained to sit in a primate chair and to perform a visual fixation task during scanning. A primate chair was used to position the animal in the magnet. Animals were motivated to engage in the task through fluid control at levels that do not affect animal physiology and have minimal impact on psychological wellbeing (Gray et al. 2016)

### Window Duration Characterization Using Stimuli with Varying Spectral Flux

Spectral flux is one of the dimensions of timbre defined as a rate of change of spectral energy (McAdams and Cunible 1992). We used stimuli in which spectral flux was characterized by the Pearson product–moment correlation (denoted as *r*_1_), henceforth termed as “correlation” between the amplitude spectra of adjoining time-frames as in equation (1).

(1) \begin{equation*} {r}_1(k)=\left(\frac{1}{s_k\bullet{s}_{k+1}}\right)\bullet \frac{1}{n}\sum_{j=1}^n\left(\left({a}_{j,k}-{\overline{a}}_k\right)\bullet \left({a}_{j,k+1}-{\overline{a}}_{k+1}\right)\right). \end{equation*}

In equation (1), ${r}_1$ is the Pearson product moment correlation between adjacent frames $k$ and $k+1$ whose amplitude spectra is denoted as ${a}_{j,k}$ for the amplitude (expressed in dB) of the $j$th frequency of $n$ such frequency components belonging to the $k$th frame, while ${\overline{a}}_k$ denotes the mean and ${s}_k$ denotes the standard deviation (SD) of the amplitude spectra corresponding to the $k$th frame.

Spectrotemporal correlation as defined above has an intuitive inverse relationship with spectral flux. As the correlation increases, the amplitude spectra of adjacent frames vary less, and spectral flux decreases. Consider a stimulus with a correlation value of one. The spectral flux here is zero since there is no change in the acoustic energy over time. For a stimulus with a correlation value of zero, the spectral flux is highest due to marked changes in spectral over time. In this experiment, we constrained spectral flux within the range zero and one.

(2) \begin{align*} {r}_n={\left({r}_1\right)}^n;\kern0.5em \mathrm{wi}{\mathrm{n}}_{\mathrm{len}}=\mathrm{fram}{\mathrm{e}}_{\mathrm{dur}}\bullet \frac{\ln \left({r}_{\mathrm{min}}\right)}{\ln \left(\left|{r}_1\right|\right)};{r}_{\mathrm{min}} \nonumber \\ =0.2;\mathrm{fram}{\mathrm{e}}_{\mathrm{dur}}=20\ \mathrm{ms}. \end{align*}

The correlation between any two frames in a stimulus is characterized by the number of frames between them and the correlation between adjacent frames. Equation (2) describes the correlation between two frames, denoted as${r}_n$, as a function of the spectrotemporal correlation ${r}_1$ between adjacent frames and the temporal distance between the frames, denoted as $n,$ when the selected frame is *n* frames away from the reference frame. This equation (Overath *et al.* 2008) also determines the length of a time window (denoted as $\mathrm{win}\_\mathrm{len}$) required to reach a minimum level of correlation (denoted as ${r}_{\mathrm{min}}$) between any two frames within it, or alternatively, the correlation between farthest frames contained within the window. The window duration is a function of the correlation ${r}_1$ and${r}_{\mathrm{min}}$ and the duration of a frame is denoted $\mathrm{as}\ \mathrm{frame}\_\mathrm{dur}$.

Intuitively, equation (2) allows the characterization of window lengths within which there is a defined degree of spectral change in the stimulus in order to define preferred brain processing as a function of optimal window length.

### Spectral Flux Stimuli

Sound stimuli were created using scripts written in MATLAB (MathWorks) version 7.1 at a sample rate of 44.1 kHz and 16-bit resolution. The amplitude spectrum was defined in terms of frames of 20-ms duration. Each synthetic stimulus was synthesized using 20 sinusoids (i.e., *n* = 20) chosen randomly from a pool of 101 logarithmically spaced frequencies between 246 and 4435 Hz. This frequency range was defined (Overath *et al.* 2008) to encompass the critical range of the human audiogram for speech perception (Niederjohn and Mliner 1982). The most sensitive part of the macaque audiogram is similar to the one found in humans (Jackson et al. 1999). Linear spline interpolation was applied to amplitude transitions between frames to avoid sudden amplitude jumps. The rise time and fall time for each sound stimulus were set at 20 ms. The mean and the SD of the amplitude spectra were set at 65 and 10 dB-rms, respectively.

The parameters in the study by Overath et al. (2008) were chosen to encompass the range of time windows between phonemes (20 ms) and syllables (300 ms) (Rosen 1992). This choice allowed inference about mechanisms relevant to the time windows for speech. The range of time windows chosen is also behaviorally relevant to macaques (Cohen et al. 2007). The *r*_min_ was set at 0.2. The correlation ${r}_1$ for each stimulus was fixed as one of five different values: 0.0 (high flux), 0.3, 0.6 (medium flux), 0.8, and 0.9 (low flux) corresponding to window durations: 20, 27, 63, 144, and 306 ms.

**Figure 2:**
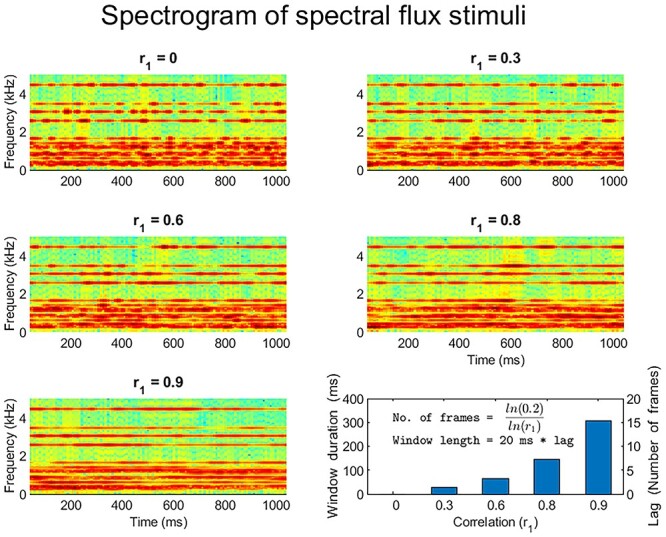
Spectrogram of exemplar stimulus from each of five different spectral flux levels employed in this study, showing the degrees of energy fluctuation from a high rate at *r*_1_ = 0.0 to a low rate at *r*_1_ = 0.9. The relationship between the spectrotemporal correlation *r*_1_ and the duration of the window to achieve a minimum correlation between any two frames within it is shown in the bottom right plot (inset formulae).

Figure 2 provides a visual representation of the spectrotemporal decomposition of exemplars of the various spectral flux stimuli employed in this study. Supplementary Files S1–S3 are example sound stimuli that correspond to *r*_1_ of 0.0, 0.6, and 0.9. The 225 different exemplars of the spectral flux stimuli were generated offline covering all conditions.

### Tonotopy Stimuli

Tonotopic information from each individual animal was used in the parcellation of function-anatomical areas of the auditory cortex. For characterizing tonotopy using the blood oxygen level–dependent (BOLD) response to spectral frequencies, sound stimuli were random-phase narrowband noise with three different pass-bands, 0.5–1, 2–4, and 8-16 kHz. The carriers were amplitude modulated (AM) with a sinusoidal envelope of 90% depth at 10 Hz to achieve a robust response in the auditory system.

### Stimulus Presentation

To record data from the auditory system that is devoid of activity due to the high-intensity noise generated by the MRI scanner, a “sparse temporal” design is utilized. With the use of a pseudo-random sequence, each adjacent trial was ensured to have a different spectral flux sound stimulus. The duration of each sound stimulus was 6 s. This duration is sufficient for the BOLD response in the macaque auditory cortex to reach a plateau (Baumann et al. 2010).

The onset and offset of the stimuli were smoothed by a linear ramp of 50 ms. The sound stimuli were presented to the monkey at an RMS sound pressure level (SPL) of 75 dB using custom adapted electrostatic headphones based on a Nordic NeuroLab system (NordicNeuroLab). These headphones feature a flat frequency response up to 16 kHz and are free from harmonic distortion at the applied SPL. SPL was verified using an MR-compatible condenser microphone B&K Type 4189 (Bruel&Kjaer) connected by an extension cable to the sound-level meter Type 2260 (same company).

### Task during Imaging

The monkey performed visual fixation on a fixation point presented in the center of a visual display in front of the animal during the entire time the sound stimulus was presented. This simple task ensured that the levels of attention remained consistent across the entire session. Moreover, it minimized the body movement of the animal by alleviating potential waiting-/boredom-related stress. The eye position was monitored at 60 Hz with a tracking (camera-based with infra-red illumination) of the pupil using iView software (SMI, www.smivision.com). The position, *X* and *Y* coordinates, of the pupil was communicated to the Cortex—a stimulation control software, for rewarding based on task performance. The task was to fixate on a target (small red square) positioned at the center of a screen when the eye trace entered within a window of fixation (~5° centered on the target) a timer started and the fixation target turned green. A continuous visual fixation (no saccades) of a randomly defined duration of 2–2.5 s was rewarded immediately by the delivery of a juice via a gravity-fed dispenser while the fixation point would disappear.

### Data Acquisition

MRI was conducted in an actively shielded 4.7 Tesla vertical scanner (Bruker Biospec 47/60 VAS) dedicated to imaging NHPs. It has an inner-bore width of 38 cm and a GA-38S gradient system from Bruker Medical. Shimming was performed with the FASTMAP algorithm (Gruetter 1993) which measures B0 field inhomogeneity to apply first- and second-order corrections to it.

Data were acquired with parallel imaging with 2-fold GRAPPA acceleration using a custom-designed (www.wkscientific.com last accessed: Sep 16, 2020) four-channel array receive coil. The RF transmission was achieved using a custom-designed saddle coil (from the same company). Both structural and functional data covered the temporal lobe and were aligned to the superior temporal plane (STP). A localizer scan helped with the slice selection.

Functional MRI measurements by BOLD contrast consisted of single-shot gradient-recalled echo-planar imaging sequences with an in-plane resolution of 1.2 mm isotropic, yielding 1.72 mm^3^ voxels and a volume acquisition time of 1.35 s. Typical acquisition parameters were as follows—time echo (TE) of 21 ms, flip angle of 90°, receiver spectral bandwidth of 200 kHz, the field of view of 9.6 × 9.6 cm^2^, with an acquisition matrix of 96 × 96 and 20 slices. A sparse design was employed where the acquisition of each volume was separated by a 10-s repetition time (TR) gap. This TR duration was necessary and sufficient to avoid recording the BOLD response to the gradient noise of the previous scan (Baumann et al. 2010).

The 6-s long stimuli were presented just before the volume acquisition where the volume was acquired at the last 1.35 s of the trial. The timing was based on previous characterization of BOLD response time course in the auditory system of macaques (Baumann et al. 2010). For every five volumes acquired with acoustic stimulus, three volumes were acquired where no stimulus was presented to obtain data for a silent baseline. In each session of 1-h duration, 360 volumes were acquired, resulting in 225 volumes for all stimuli or 45 volumes per each of 5 stimulus levels while 135 volumes correspond to silence. Data from monkey M1 were collected over five sessions (thus, 225 volumes were obtained for each stimulus level), data from monkey M2 were collected over four sessions (thus, 180 volumes were obtained for each stimulus level), while data from monkey M3 were collected over six sessions (thus, 270 volumes were obtained for each stimulus level).

A structural scan was acquired at the end of each functional scanning session. Anatomical MR images are T1-weighted (T1w) images, consisting of a 2D magnetization-prepared rapid gradient-echo sequence with a 180° preparation pulse, TR = 2000 ms, TE = 3.74 ms, TI = 750 ms, 30° flip angle, receiver bandwidth = 50 KHz, and an in-plane resolution of 0.67 × 0.67 mm^2^ with a slice thickness of 0.6 mm. These structural scans cover the same field of view as the functional scans.

### Data Analysis

MR images were first converted from scanner’s native file format into a common MINC file format, 3D for the anatomical data and 4D (*x*, *y*, *z*, and *t*) for the functional data, using the Perl script pvconv.pl available online (http://pvconv.sourceforge.net/ last accessed: Sep 16, 2020). From MINC format, it was converted to NIfTI file format standard using MINC tools. These raw fMRI data were processed using Statistical Parametric Mapping (SPM12) software (www.fil.ion.ucl.ac.uk/spm last accessed: Sep 16, 2020) using MATLAB 7.1 software.

In the preprocessing steps, first, rigid body motion compensation was performed. Next, image volumes from multiple sessions were combined by realigning all volumes to the first volume of the first session. Then, these data were spatially smoothened using a Gaussian kernel with full-width at half-maximum of 3 mm. A standard SPM regression model was used to partition components of the BOLD response at each voxel. The five conditions, each of five different spectrotemporal correlation values, were modeled as effects of interest compared with silent baseline and their stimulus onsets were convolved with a canonical hemodynamic response function. Next, the time series was high-pass filtered with a cut-off of 120 s to remove low-frequency signal drifts mainly due to scanner instabilities. Finally, these data were adjusted for global signal fluctuations also known as global scaling to account for differences in system responses across multiple sessions.

In a general linear model analysis of the combined sessions, which included the motion parameters, the voxel-wise response estimates the regression coefficients (denoted beta). The *t*-values for the contrast of the different stimuli versus the silent baseline were also calculated. The data were masked retaining voxels with significant values for the combined stimuli versus silent baseline (*P* < 0.001, uncorrected for multiple comparisons across the auditory cortex).

### Best Frequency Tonotopy Map

Data for the tonotopy experiment were acquired from the monkeys after data for the main spectral flux experiment were acquired. Tonotopy data using three frequency bands (0.5–1, 2–4, and 8–16 kHz) were collected from monkey M1 over two sessions (135 volumes per frequency band in total) and from monkey M2 over one session (150 volumes per frequency band in total). No tonotopy data were collected in monkey M3.

Map of preferred response to different frequency bands is known as “best-frequency map.” This map was calculated by identifying, voxel by voxel, which of the frequency conditions showed the highest beta, that is, regression coefficient. This map was computed in each animal across all voxels whose sound versus silence contrast was significant (*T* > 3.1, *P* < 0.001 uncorrected for multiple comparisons across the auditory cortex). The resulting map represents the preferred frequency for each voxel.

The BOLD activation associated with sound stimulation was analyzed in voxel space. Sound related activation (*P* < 0.001 uncorrected for multiple comparisons across the auditory cortex) was observed in the STP, which had a generally symmetrical pattern across the hemispheres. Best-frequency maps showed well-established mirror symmetric high–low–high frequency gradients across the auditory core and belt regions bilaterally (Merzenich and Brugge 1973; Morel et al. 1993; Kosaki et al. 1997; Rauschecker et al. 1997; Bendor and Wang 2008; Baumann et al. 2013; Joly et al. 2014; Baumann et al. 2015; Poirier et al. 2017). Parcellation of the auditory cortex in macaques into various regions of interest (ROIs) was achieved using a combination of best-frequency maps from tonotopy experiments and high-resolution T1 and T2 images (Joly et al. 2014).

### Parcellation

To map the auditory subfields, information from tonotopy fMRI data, macro-anatomical features (cortical folding), and anatomical MRI were combined. The ratio (Joly et al. 2014) of T1w and T2w images provided an index that represented average intensities across the cortical thickness. Highest values of T1/T2 ratio indicated gray matter voxels and were used to identify the location of A1 and R fields. The boundary between A1 and R was identified via the frequency reversal occurring between these regions in the best frequency map of the tonotopy experiment since the posterior end of A1 and anterior end of R prefers high frequency while the anterior end of A1 and the posterior end of R, that is, boundary prefers low frequency. To overcome the similarity of frequency preference between core and belt regions and the difficulty in parcellation of medial belt regions, the T1/T2 ratio is utilized to demarcate between core and belt since this ratio is high in the core regions but lower in the belt regions.

**Figure 3:**
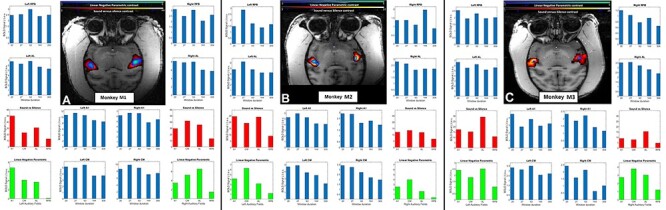
Contrast for the negative parametric effect of time-window duration and sound versus silence contrast in three monkeys. Linear negative parametric contrast (bluish-green hue) is overlaid on sound minus silent baseline contrast (reddish-yellow hue). Both contrast maps are rendered on top of an axial section passing through auditory cortex (T1 structural scan) in the STP in (*A*) Monkey M1, (*B*) Monkey M2, and (*C*) Monkey M3. Histograms in blue show the BOLD signal for each of the five conditions as a function of different ROIs: core—A1, medial belt—CM, lateral belt—AL, and parabelt—RPB. Histograms in red and green summarize the sound versus silence and negative parametric contrast as a function of these selected auditory cortical regions. The thresholds on statistical maps were kept at *T* > 3.1 or *P* < 0.001 uncorrected for multiple comparisons across the auditory cortex. Sound versus silence contrast shows that this synthetic stimulus employed in this study robustly activated most auditory cortical areas bilaterally. A negative linear parametric contrast (implies BOLD decreases with increasing time windows) is seen in the auditory core and belt regions bilaterally.

The exact method and tools used in parcellation are described here. The subject-specific parcellation of the auditory cortical subfields follows the scheme reported in Reveley et al. (2017). The original atlas was used as provided in the registered format, with the population MRI primate brain template published in Seidlitz et al. (2018) and available at https://github.com/jms290/NMT (last accessed: Sep 16, 2020). For each monkey, information from the tonotopic mapping from bold-weighted functional MRI data, macro-anatomical features (cortical folding of the lateral sulcus), and anatomical MRI were combined. The lateral fissure was used to run a (local) surface-based coregistration from the NMT template to the subject-native space in order to initialize the registration, then nonlinear registration was further computed with alignment of the antero-posterior border between A1 and R to the first reversal from high–low–high frequency reversal from the tonotopic mapping (Joly et al. 2014) using 3D Slicer (ITK-based registration framework, www.slicer.org last accessed: Sep 16, 2020). Lastly, the final local lateral adjustment of the full parcellation was applied to overlap the *x*-coordinate of the center of the core regions (especially A1/R) to the peak location (within the gray matter) of the T1w-bias corrected map (Geyer et al. 2011; Glasser and Van Essen 2011; Joly et al. 2014).

Thus, the following fields were identified in each hemisphere in each monkey M1 and M2, namely, A1, AL, CL, CM, CPB, ML, R, RM, RPB, RT, RTL, RTM, RTp, STGr, and Tpt. We could not collect tonotopy data in monkey M3, and parcellation is based solely on macro anatomical features (cortical folding of the lateral sulcus) identified combined with the anatomical MRI of the animal.

### Window Duration Preference

To reveal the spatial organization of window duration preference, a contrast map was generated by projecting the functional data of the acquired volumes onto the anatomical scans. Next, the response strength of the shorter time windows (or lower spectrotemporal correlation) was contrasted with the longer time windows (or higher spectrotemporal correlation). This contrast map was calculated voxel by voxel by summing the differentially weighted regression coefficients (beta) of the various spectrotemporal correlations. The contrast maps obtained using the following weights (2, 1, 0, −1, and −2) are henceforth referred to as “linear negative parametric” contrast. The negative parametric contrast represents the degree of preference for shorter over longer time window duration (or, alternatively, low over high spectrotemporal correlation levels).Though Overath et al. (2008) used an exponential decay contrast in the main analysis, they showed in supplementary analysis that the results do not differ between the linear negative parametric and exponential decaying contrasts. We report the results from linear negative parametric contrast, although we also verified that the results when using exponential decaying contrast are similar.

This linear negative parametric contrast map was subject to small volume correction for multiple comparisons where the small volume was the auditory cortex defined by sound versus silence contrast (with cluster defining threshold at *P* < 0.001 and appropriate cluster correction for multiple comparisons).

## Results

### Activation to Sound

In the main experiment on time windows, the fMRI BOLD response was recorded across the entire auditory cortex to sound stimuli with five different spectrotemporal correlations. These stimuli corresponding to varying degrees of spectral flux were presented to three macaques undergoing fMRI. The BOLD activation associated with sound stimulation was analyzed in voxel space. Sound related activation (*P* < 0.001 uncorrected for multiple comparisons across the auditory cortex) was observed in the STP that had a generally symmetrical pattern across the hemispheres. This synthetic spectral flux stimulus robustly activated cortical areas bilaterally (Fig. 3).

**Figure 4:**
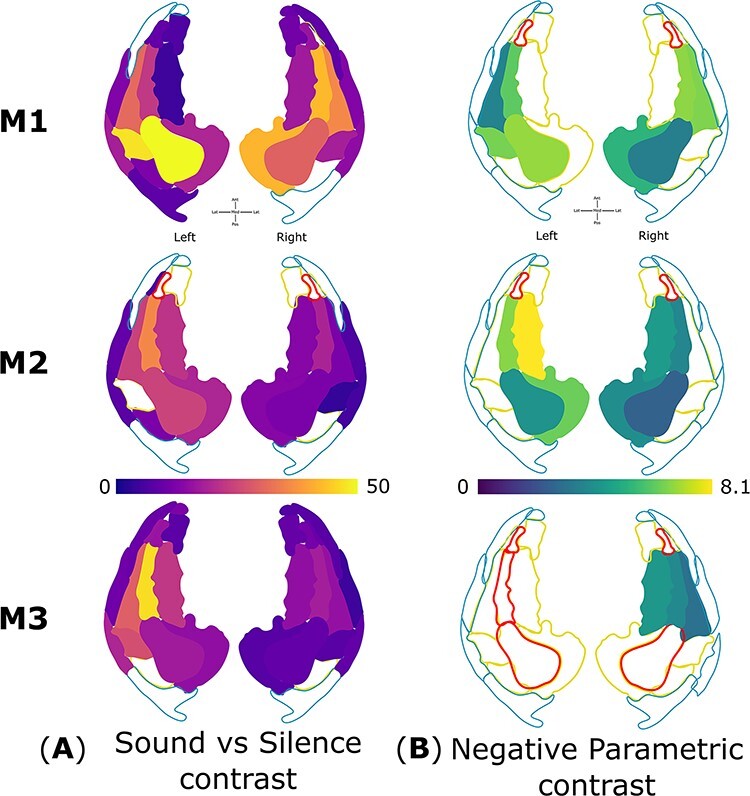
Visual representation of sound versus silence contrast and linear negative parametric contrast betas across various ROIs of three macaques. The auditory ROI are color-coded individually for sound minus silent baseline (reddish-yellow) and linear negative parametric contrast (greenish-yellow) in each hemisphere of monkeys M1, M2, and M3. (*A*) Sound versus silence contrast panel shows that the synthetic spectral flux stimulus robustly activated most auditory cortical areas bilaterally. (*B*) Negative parametric contrast panel shows that BOLD decreased with increasing time-window duration in the auditory core and medial belt regions bilaterally.

**Figure 5:**
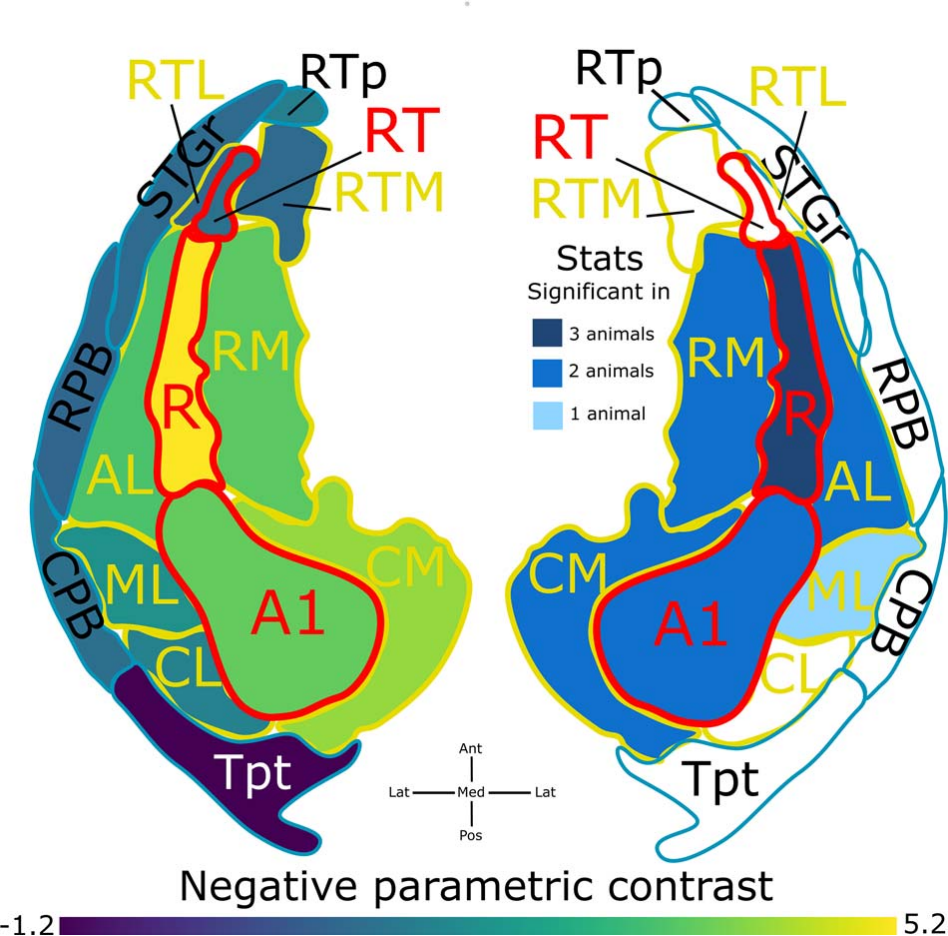
Visual summary of the results from three monkeys. Group average and summary of statistics on linear negative parametric contrast evaluated across three monkeys in each ROI across the auditory cortex combining across hemispheres. ROIs on the left are color-coded to reflect the average value, while ROIs on the right are color-coded to reflect the number of animals in which the result is significant.

### Window-Duration Preference

The contrast maps for monkey M1, M2, and monkey M3 are shown in Figure 3 as blue-green hue (linear negative parametric contrast) overlaid on auditory activation in red-yellow hue. The results from linear negative parametric contrast survive small volume correction for multiple comparisons across auditory cortex as defined by sound versus silence contrast (with cluster defining threshold at *P* < 0.001 and appropriate cluster correction for multiple comparisons) in both hemispheres of monkeys M1 and M2 but only in the right hemisphere of monkey M3. The data demonstrate that BOLD increases with decreasing time windows across all four auditory regions (A1, CM, AL, and RPB) on either side as demonstrated by the linear parametric effects plotted in green in Figure 3. The plots of BOLD as a function of window length in these areas were similar on the two sides. In other words, BOLD is highest for shorter time windows in all three monkeys.

### ROI-Based Analysis

Using the MarsBaR toolbox (version 0.44) (Brett et al. 2002), the sound versus silence contrast and linear negative parametric contrast within each ROI (estimated earlier) was averaged across all voxels where sound versus silence contrast was significant (T > 3.1, *P* < 0.001 uncorrected for multiple comparisons across the auditory cortex). ROI-level statistical threshold was applied at *P* < 0.05, corrected for multiple comparisons (*n* = 30 ROIs) in a given animal. Figure 4 visualizes these data as a function of ROIs in the three monkeys. Supplementary Tables S1 and S2 provide the beta and significance values for sound versus silent baseline contrast and linear negative parametric contrast across various ROIs in the three monkeys. We observe that the sound-driven activation is robust in core and belt cortical areas bilaterally but not in parabelt tertiary areas. Further, the preference for short windows as conveyed by the linear negative parametric contrast was present across most ROIs of core and belt areas in five hemispheres of three animals. The representative beta value in each ROI was then averaged across hemispheres of all three animals. Figure 5 summarizes the group mean and number of hemispheres in which the linear negative parametric contrast is statistically significant. Auditory core areas showed a strong preference for shorter time windows. The belt areas continued to prefer shorter windows, though the preference was less strong. Further, BOLD did not change systematically with time windows in parabelt regions which could be also due to a lack of response to sounds in these tertiary areas.

**Figure 6:**
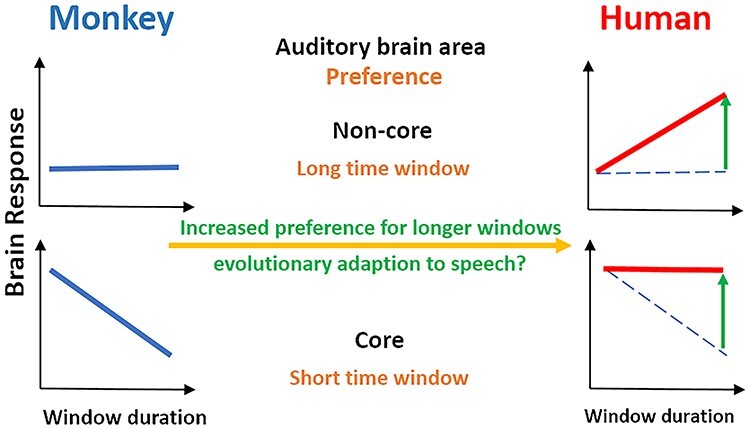
Summary of time window preference in monkeys and humans. The top panels represent noncore auditory cortex, while bottom panels represent core auditory cortex. Monkey auditory cortical preference (shown in blue lines) in the core areas is for short time windows, but there is gradual reduction in this preference as we progress to noncore areas. Human auditory cortical preference (shown in red lines) in noncore homologs is for long time windows, while there is a reduction in this preference in core homologs. In essence, core areas relatively prefer short time windows and this preference relatively shifts toward long time windows in noncore areas in both humans and monkeys. So, the anatomical organization of time-window processing is similar across primates despite the increased preference (shown in green arrows) for longer time windows across all cortical areas in humans as compared with monkeys (shown in blue dotted lines). This specialization of the human brain for processing longer durations of acoustic stimuli could be an evolutionary adaptation to process speech.

## Discussion

This work examined the anatomical organization of time-window processing using synthetic broadband spectral flux stimuli (Fig. 2) in macaques. We aimed to test common principles related of time-window processing in primates and to seek differences in the specific organization in the two species that reflect natural listening. The stimuli had systematic variation in the spectral correlation over time and related variation in the optimal time window of analysis, allowing us to map responses to changes in time windows for analysis in different parts of auditory cortex.

This experiment investigated the differences in the BOLD signal as a function of the time-window duration in the macaque auditory core, belt, and parabelt regions. Using synthetic stimuli, we observed a preference for shorter time windows (higher BOLD response for short windows) in auditory core and to a lesser extent in belt regions bilaterally in monkeys. Using the same stimuli, a previous human study (Overath et al. 2008) did not report a preference for any specific time window in bilateral HG (no parametric effect) but reported a preference to longer time windows (i.e., BOLD is highest for long windows) in auditory association cortex and right STS in humans. The current data show a difference in the preference for time windows in the auditory cortex of macaques and humans. We speculate that these species differences are related to the differences in the perception of temporal windows between species.

There is behavioral evidence for differences between humans and macaques in the auditory temporal analysis. O'Connor et al. (2011) have compared sensitivity to detect sinusoidally varying AM tones between rhesus macaques and humans. In humans, they found peak sensitivity to detection of AM that ranged between 10 and 30 Hz modulation rates depending on tone duration, while for macaques, the peak sensitivity ranged between 40 and 100 Hz. Further, they reported a greater sensitivity in humans over macaques for detection of AM noise at lower modulation rates (<10 Hz). This suggests a greater preference for slower temporal rates in humans.

Itoh et al. (2019) recorded electroencephalography from humans and macaques scalp while passively presenting them with pure tones of varying duration (2–200 ms). They analyzed how the P1-N1-P2-N2 complex diminished in amplitude as the sound duration decreased, suggesting their underlying window of temporal integration. They reported elongation of time window of integration in the later stages of human auditory cortical processing compared with macaques, which is entirely consistent with our findings. The current study allows us to identify time-window preferences in core and belt with anatomical precision.

Recent work has investigated the cortical encoding of natural sounds using fMRI in humans and made a comparison with macaques using identical stimuli and identical modeling. The temporal modulation function in macaques shows a preference for faster modulation rates, with its peak >30 Hz (Erb et al. 2019), while the preference in humans is for slower modulation rates centered at 3–4 Hz (Santoro et al. 2014, 2017). These studies support differences in preference for temporal modulation rate between primates.

There is also neurophysiological evidence to support the behavioral results. Niwa et al. (2012a) recorded from single units (SUs) and multiunits (MUs) in the A1 of behaving rhesus macaques, which were detecting AM in broadband noise. They reported that macaques are most sensitive to detect AM rates between 30 and 120 Hz, which is in agreement with prior macaque behavioral experiment (O'Connor et al. 2011). They further reported that the neurons (SUs and MUs) whose neural threshold for AM detection exceeded behavioral thresholds mostly had their best modulation frequency (BMF) in the above described most sensitive to AM range and exhibited nonsynchronous responses. On this same data, Niwa et al. (2012b) reported that the rate-BMF ranged between 15 and 120 Hz for MU and between 15 and 250 Hz for SUs. Though earlier results (Malone et al. 2007; Scott et al. 2011; Yin et al. 2011; Johnson et al. 2012; Overton and Recanzone 2016) in A1 of awake nonbehaving macaques show neuronal peak response for AM rates (up to 20 Hz), which is slower than behavioral results (O'Connor et al. 2011), it is consistent with these results in behaving macaques (Niwa et al. 2012a, 2012b). This shows that neurons in macaque A1 respond best to AM rates faster than those observed in human behavior. There is evidence to suggest this is generic to other NHPs as well since Liang et al. (2002) recorded from single neurons in A1 of awake nonbehaving marmoset monkeys in response to AM tones and reported that the modulation frequency at which neurons are maximally sensitive is at 16–32 Hz AM rate, which is higher than human behavior.

Thus, certain behavioral data (O'Connor et al. 2011), neurophysiological data in behaving animals (Niwa et al. 2012), and BOLD data (Erb et al. 2019) support our findings of an increased preference toward faster rates or shorter time windows in monkeys over humans.

In our data, we observe a relative reduction in preference to short time windows as we progress from core to belt and parabelt. Similarly, Niwa et al. (2013) who recorded from ML and A1 areas, reported that though rate coding for AM in ML is similar to A1, phase-locking for AM rates >=15 Hz is worse. This suggests that noncore cortical areas in macaque respond to relatively lower AM rates than A1. Bieser and Muller-Preuss (1996) recorded responses to AM tones from SUs in the auditory cortex of awake nonbehaving squirrel monkey and reported that the BMF in A1 was 8–16 Hz AM rate, while noncore fields like AL failed to follow the AM envelope. These NHP studies suggest a broadening of time windows as one progressed from core to noncore areas in the AC.

A number of previous studies in humans and macaques (Fig. 1) have examined the mechanisms for analysis of temporal structure of sounds. The majority of these suggest a uniform preference for longer time windows (>100 ms duration) across human auditory cortex in both primary and nonprimary regions compared with NHPs (<100 ms duration). There is evidence to support this kind of specialization. Modulation at 3–10 Hz (100–333 ms window) seems critical for the processing of spoken syllables and speech intelligibility (Luo and Poeppel 2007). This suggests a possible reason for observing increased preference for longer time windows in humans. Cohen et al. (2007) reported high variance between macaque vocalization categories at higher temporal modulation frequencies between 5 and 20 Hz (upto 50 ms), which are very relevant for categorization of vocalizations. Joly et al. (2012) reported that certain macaque vocalizations have very high temporal modulation rates when compared with human speech. These studies suggest a possible reason for observing increased sensitivity to shorter time windows (<100 ms duration) in monkeys. Thus, this need to process speech in humans and vocalizations in macaques might account for the differences in the sensitivity for temporal processing rates between humans and monkeys. Thereby, the tuning of the auditory cortex to syllabic rate (i.e., a long time window) might be unique to humans and possibly an outcome of divergent evolution in humans alongside the development of speech.

The increased sensitivity toward longer time windows observed in humans in the previous study (Overath et al. 2008) might be due to species differences in the preferred window of temporal integration. Figure 6 shows a schematic conveying the auditory cortical organization of time-window processing in primates, namely, core areas relatively prefer short time windows and noncore areas relatively prefer long time windows. This anatomical organization of time-window processing is similar across primates despite the overall tuning of human auditory cortex for longer time windows due to speech processing. Such anatomical organization of intrinsic timescales have been previously reported (Murray et al. 2014) in other sensory cortices and frontal areas of monkeys.

We speculate that the increased preference for a longer time window of integration in human auditory cortex reflects specialization for perception of syllabic rates of speech at (2–8 Hz) produced by humans as discussed in the Introduction. By contrast, monkey vocalizations do not show this preferred range, reflecting differences in their vocal tract, brain mechanisms that control it (Fitch et al. 2016; Belyk and Brown 2017; Fischer 2017). So, we speculate that the differences in the preference for auditory time windows might have arisen as an evolutionary adaptation to speech in humans.

## Conclusion

To summarize, we hypothesize a similar anatomical organization of time-window processing in macaques and humans which demonstrates a gradient of preferred responses that changes from core to belt cortex (or the human homologs of these). Macaques show a preference for short time windows in core areas and no preference in higher areas. Humans show no preference for short time windows in core areas and a preference for longer time windows in higher areas. This preference for the analysis of long time windows in humans provides a mechanism for the preferential analysis of syllabic rates of human speech.

## Supplementary Material

supplementary_file_1_bhab434

supplementary_file_2_bhab434

supplementary_file_3_bhab434

Auditory_time_windows_processing_in_primates_Supplementary_text_bhab434

## Notes

The MATLAB-based source code for acoustic stimulus generation, NIH-CORTEX-based fMRI data acquisition scripts, macaque fMRI data, and SPM toolbox-based data processing scripts in MATLAB are made publicly available at the following repositories: https://doi.org/10.17605/OSF.IO/ANQ4P and https://doi.org/10.17605/OSF.IO/D2BZ7. *Conflict of Interest*: The authors disclose that there is no conflict of interest.
