# Supplementary material for: The representation of time windows in primate auditory cortex
Source: Cereb Cortex. 2021 Dec 7;32(16):3568–80. doi: 10.1093/cercor/bhab434 (PMC9376871; doi:10.1093/cercor/bhab434)
Supplement: Auditory_time_windows_processing_in_primates_Supplementary_text_bhab434 [file auditory_time_windows_processing_in_primates_supplementary_text_bhab434.docx]

# The representation of time windows in primate auditory cortex

**Supplementary text - Literature survey**

The studies in humans and non-human primates that are included in the literature survey plot (Figure 1 in main text) are briefly discussed here.

Zatorre RJ and P Belin (2001) used PET in humans with a synthetic stimulus, in which temporal complexity was varied by changing the rate of temporal switching between two tones from the slowest rate or longest time window at 667 ms to the fastest rate or shortest time window at 21 ms. They showed a preference for shorter time windows (21 ms) in auditory core homologues as well as in anterior superior temporal gyrus (STG) bilaterally while no areas were demonstrated to prefer longer time windows. Jamison HL et al. (2006) employed the same stimulus as in Zatorre RJ and P Belin (2001) with sparse fMRI but with only extreme values of temporal rates corresponding to windows of 21 ms and 667 ms. The shorter window (21 ms) was associated with increased bilateral activity in Heschl’s gyrus (HG). The response to increased temporal variation was greater in the left than the right posteromedial HG. Both of these studies differ from the general consensus in that they do not report a preference for longer windows in antero-lateral auditory cortex.

Schönwiesner M et al. (2005) manipulated temporal complexity using stimuli that differ in modulation rate, but not in bandwidth or energy. This study used a narrowband stimulus comprising 3 spectral components varying in temporal modulation rates from 5 Hz to 30 Hz (200 ms to 33 ms windows). Sparse fMRI demonstrated an increased preference for shorter time windows (33 ms) in non-primary cortex in the left superior temporal gyrus (STG). No-differential sensitivity to specific windows was demonstrated in HG on either side. No preference for longer time windows was demonstrated anywhere on the superior temporal plane or STG.

Giraud AL et al. (2000) used sinusoidally amplitude-modulated broadband noise with rates from 4 to 256 Hz, corresponding to a windows of 4 to 250 ms with continuous-acquisition functional magnetic resonance imaging (fMRI) in humans. The data demonstrate longer preferred time windows in both primary (125 ms) and non-primary cortex (250 ms).

Liegeois-Chauvel C et al. (2004) used sinusoidally amplitude-modulated white noise with rates from 4 to 128 Hz, corresponding to a window of 8 to 250 ms with electrocorticography (ECoG) in humans. The data demonstrate preference for longer time window in posteromedial HG (250 ms) and anterolateral HG (250 ms on left and 125 ms on right).

Boemio A et al. (2005) used synthetic stimuli generated by concatenating narrow band noise of different segmental durations, from 12 to 300 ms (spanning the range between segmental transitions to syllables in speech). Single-trial sparse fMRI showed a preference for longer time windows (300 ms) in bilateral HG, STG, and superior temporal sulcus (STS).

Frequency Following Response to periodic click trains is a measure of temporal synchrony which belies the underlying temporal window of integration employed by a cortical area. Brugge JF et al. (2009) recorded responses to periodic click trains from Heschl’s Gyrus in humans using ECoG. They reported (see Fig 5) peak temporal synchrony at 200 Hz click train (time window of 5 ms) in posteromedial HG (Nourski KV et al. 2013) while there are no temporal synchrony in anterolateral HG.

Schonwiesner M and RJ Zatorre (2009) used dynamic ripple stimuli with ripple velocities ranging from 1.6 to 27.2 Hz corresponding to a time window of 37 to 625 ms. Using high-resolution fMRI in humans they reported (see Fig 3) that medial HG preferred a long time window (270 ms or 3.7 Hz temporal rate) and planum polare also preferred a long time window (322 ms or 3.1 Hz temporal rate).

Barton B et al. (2012) used amplitude-modulated broadband (0 to 8 kHz) noise with modulation rates of 2 to 256 Hz corresponding to window durations of 4 to 500 ms. Using a travelling wave method, modified to allow sparse-sampling on fMRI, and applying neither spatial nor temporal smoothing, nor motion correction, a preference for faster modulation rates (or short windows of 15 ms) was shown in medial HG and a preference for slower modulation rates (or long windows of 250 ms) in lateral HG.

Overath T et al. (2012) used sinusoid, narrow-band and broad-band noise amplitude modulated at rates from 3 to 57 Hz corresponding to a time window of 17 ms to 333 ms. Using fMRI in humans, they reported (see Fig 2) preference to long time windows (333 ms) in both medial HG and lateral HG.

Herdener M et al. (2013) used broadband (25 Hz to 8 kHz) noise that was amplitude modulated at rates of 2 to 32 Hz corresponding to window durations of 31 to 500 ms. Sparse fMRI demonstrated a preference for faster modulation rates (or short windows of 31 ms) in medial HG and preference for slower modulation rates (or long windows of 250 ms) in lateral HG.

Leaver AM and JP Rauschecker (2016) used amplitude modulated white noise with modulation rates of 1.33 to 52 Hz corresponding to window durations of 19 to 750 ms. They employed fMRI in humans and reported (see Fig 5) preference to longer time windows (120 ms) in both medial and lateral HG.

Belin P et al. (1998) used pseudo-speech syllables to examine the processing of formant transitions of different durations, namely ‘rapid’ (40 ms) and ‘extended’ (200 ms). The study is distinct from the other studies discussed here in using a stimulus derived from natural speech. Positron emission tomography (PET) in humans showed a lack of differential activations of the left auditory cortex to the different durations while the right auditory cortex demonstrates sensitivity to the longer windows (200 ms).

Santoro R et al. (2014) have used natural sounds including human speech and non-speech sounds, musical instruments, environmental sounds and animal cries. Analysis was based on examination of the modulation content of the auditory stimuli determined by a bank of 2D modulation-selective filters, tuned to temporal modulation frequencies ranging from 1 to 27 Hz corresponding to time windows between 37 ms to 1s. The work supported the encoding of coarse spectral information with high temporal precision (requiring a relatively shorter analysis window) in postero-medial areas, and the encoding of fine spectral information requiring longer analysis window in antero-lateral auditory areas (lateral belt homologues).Santoro R et al. (2017) assumed that the reconstruction accuracy for natural sounds reflects the tuning properties of underlying neuronal populations, they found that entire auditory cortex is tuned to 3.1 Hz temporal modulation (322 ms time window).

Overath T et al. (2015) used sound quilts constructed by shuffling segments, ranging from 30 to 960 ms of natural sounds, approximately preserving its properties on short timescales while disrupting its properties on longer timescales. If two different sound quilt durations both exceed the time window of analysis pertaining to a cortical region’s characteristic neuronal receptive fields then the corresponding responses would be similar as they would be similarly natural from the perspective of receptive field. So the shortest quilt duration to which the highest BOLD response was obtained in a cortical area would reveal its underlying duration of analysis time window. They employed fMRI in humans and reported (see Fig 8) preference to long time windows (480 ms) in both posteromedial and anterolateral auditory cortex.

*Non-human primates*

Steinschneider M et al. (1998) recorded responses to click trains ranging 10 Hz to 500 Hz corresponding to time windows of 2 to 100 ms from multi-units in A1 of awake macaques (Macaca fascicularis) and reported (see Fig 9) temporal synchrony up to 150 Hz click train (time window of 7 ms). Similarly, Lu T et al. (2001) recorded responses to click trains with wide-band, narrow-band click trains of inter-click interval ranging from 3 to 100 ms and click trains with random inter-click-interval (ICI) from the A1 of awake marmosets and reported (see Fig 3) neurons that exhibited synchronized responses to smallest ICI of 3 ms.

Bendor D and X Wang (2007) used acoustic pulse trains composed of narrowband tones, noise and clicks as carriers with pulse rates ranging from 4 to 48 Hz corresponding to a time window of 21 to 250 ms. They recorded from single units in A1, R, and RT auditory cortical regions of awake marmosets. They reported (see Fig 1b, Fig 7) that more neurons in A1 exhibited stimulus synchronization up to 48 Hz (21 ms time window) compared to neurons in R and RT which exhibited synchronized responses for rates less than 10 Hz (100 ms time window). They suggest that the neural representation transforms from a temporal code in the posteromedial cortical areas to a rate code in the anterolateral cortical areas. This agrees with the idea that the analysis time window widens along the caudo-rostral axis of the auditory cortex.

Bendor D and X Wang (2008) used sinusoidally amplitude-modulated tones at rate of 4 to 2048 Hz corresponding to windows of 0.5 to 250 ms. They recorded from single units in the core regions A1, R and RT regions of the auditory cortex of awake marmosets. . They reported (see Fig 14) that neurons in A1 that synchronized to AM up to 64 Hz (16 ms time window) while neurons in R and RT regions exhibited stimulus synchronization to AM up to 16 Hz (62 ms time window) . Further, a model was proposed based on a short temporal integration window in A1 that increases in the anterior regions, R and RT.

Scott BH et al. (2011) recorded from single units from the auditory cortical fields in awake macaques. Synchronization of spike discharges was sought to amplitude modulated tones at rates of 0.7 to 200 Hz corresponding to time windows of 5 ms to 1.5 s, similar to those present in macaque vocalisations and human speech. More neurons in A1 synchronize to modulation rates up to 30 Hz compared to (medial and lateral) belt areas apart from R where most neurons exhibit a synchronization for 10 Hz modulation rate. So a window of temporal integration in A1 of 33 ms was demonstrated while in R and anterior belt areas this was 100 ms. Neurons in medial belt area CM synchronized to higher amplitude modulation rates like A1 (88 Hz in CM vs 46 Hz in A1). However, neurons in medial and lateral belt area M, L synchronized to lower amplitude modulation rates like R (12 Hz in M, 11 Hz in L and 10 Hz in R).

Niwa M et al. (2013) used amplitude modulated broad-band noise with modulation rates ranging from 2.5 to 1000 Hz corresponding to a time window of 1 to 400 ms. They recorded response from single-units and multi-units in A1 and ML cortical areas. They reported (see Fig 2) that the ability of neurons in ML was generally worse at phase locking than A1 for AM rates >= 15 Hz (time window 67 ms) since A1 phase lock up to 100 Hz (10 ms time window). This suggests that a longer time window is employed in non-primary cortical areas of macaque than primary auditory cortical areas.

Baumann S et al. (2015) carried out sparse fMRI on macaques during the presentation of amplitude modulated broadband noise at rates of 0.5 to 512 Hz corresponding to a window duration of 2 ms to 2 s. The data show a preference for 128 Hz modulation rate (8 ms time window) in postero-medial areas and 2 Hz modulation rate (500ms time-window) in antero-lateral areas of the auditory cortex.

The fMRI macaque study of Erb J et al. (2019) used identical methods to the human study of Santoro R *et al.* (2014) to analyse the encoding of natural sounds in the macaque auditory cortex. The data show a preference for faster (24 Hz) characteristic temporal modulation ( 42 ms time window) in posterior auditory areas and a preference for slower (4 Hz) rates ( 250 mstime window) in anterior auditory areas, consistent with the human data.

| ROI | Monkey M1 | | | | | | Monkey M2 | | | | | | Monkey M3 | | | | | |
| --- | --- | --- | --- | --- | --- | --- | --- | --- | --- | --- | --- | --- | --- | --- | --- | --- | --- | --- |
|  | Left hemi | | | Right hemi | | | Left hemi | | | Right hemi | | | Left hemi | | | Right hemi | | |
|  | n | β | p | n | β | p | n | β | p | n | β | p | n | β | p | n | β | p |
| A1 | 42 | 50 | <1e-7 | 24 | 29.62 | <1e-7 | 21 | 24.56 | <1e-7 | 10 | 12.12 | <1e-7 | 53 | 17.18 | <1e-7 | 26 | 8.95 | <1e-7 |
| AL | 51 | 30.85 | <1e-7 | 50 | 35.65 | <1e-7 | 40 | 24.11 | <1e-7 | 20 | 11.89 | <1e-7 | 135 | 29.13 | <1e-7 | 124 | 15.47 | <1e-7 |
| CL | 8 | 12.27 | <1e-7 | 0 | - | - | 0 | - | - | 0 | - | - | 0 | - | - | 0 | - | - |
| CM | 14 | 22.95 | <1e-7 | 24 | 41.82 | <1e-7 | 8 | 19.2 | <1e-7 | 8 | 13.5 | <1e-7 | 41 | 15.16 | <1e-7 | 13 | 7.71 | 8e-6 |
| CPB | 53 | 18.09 | <1e-7 | 18 | 14.69 | <1e-7 | 16 | 8 | <1e-7 | 1 | 5.48 | 5e-4 | 121 | 19.13 | <1e-7 | 76 | 9.08 | <1e-7 |
| ML | 24 | 47.53 | <1e-7 | 8 | 17.98 | <1e-7 | 12 | 12.1 | <1e-7 | 3 | 3.6 | 2e-6 | 29 | 26.54 | <1e-7 | 11 | 7.9 | <1e-7 |
| R | 23 | 22.53 | <1e-7 | 29 | 42.39 | <1e-7 | 23 | 35.42 | <1e-7 | 24 | 18.85 | <1e-7 | 25 | 46.84 | <1e-7 | 22 | 17.78 | <1e-7 |
| RM | 1 | 4.8 | 0.024 | 27 | 17.31 | <1e-7 | 6 | 21.7 | <1e-7 | 7 | 12.98 | <1e-7 | 71 | 22.27 | <1e-7 | 59 | 15.17 | <1e-7 |
| RPB | 28 | 12.8 | <1e-7 | 22 | 13.22 | <1e-7 | 14 | 9.01 | <1e-7 | 13 | 6.64 | 5e-6 | 28 | 10.71 | <1e-7 | 3 | 4.58 | 2e-6 |
| RT | 7 | 9.03 | 6e-6 | 6 | 16.01 | <1e-7 | 0 | - | - | 0 | - | - | 23 | 16.08 | <1e-7 | 22 | 11.3 | <1e-7 |
| RTL | 5 | 9.14 | 7e-6 | 17 | 16.4 | <1e-7 | 1 | 6.78 | 1e-3 | 0 | - | - | 15 | 16.4 | <1e-7 | 12 | 9.84 | <1e-7 |
| RTM | 1 | 6.67 | 1e-3 | 7 | 10.92 | <1e-7 | 0 | - | - | 0 | - | - | 1 | 7.97 | 1e-3 | 2 | 9.32 | 6e-5 |
| RTp | 0 | - | - | 2 | 10.25 | 3e-3 | 0 | - | - | 0 | - | - | 2 | 12.3 | 3e-6 | 1 | 7.34 | 2e-3 |
| STGr | 0 | - | - | 17 | 8.84 | <1e-7 | 0 | - | - | 0 | - | - | 69 | 10.28 | <1e-7 | 30 | 7.92 | <1e-7 |
| Tpt | 4 | 8.49 | <1e-7 | 0 | - | - | 0 | - | - | 0 | - | - | 0 | - | - | 0 | - | - |

Supplementary Table S1 Sound minus silent baseline contrast details from various fields in the auditory cortex of three monkeys.

Number of voxels, beta and significance value are given for ‘sound versus silence’ contrast across all the ROIs in monkeys M1, M2, and M3. Number of voxels (n) in each ROI of monkeys whose t-statistic for ‘sound versus silence’ survive statistical threshold at a single voxel level of p<0.001, uncorrected for multiple comparisons across the auditory cortex. The beta is averaged across these surviving voxels. In some ROIs, where beta values are omitted, no voxels within this ROI survived this threshold. The significance value (p) of each ROI is corrected for multiple comparisons across ROIs within each monkey. This data indicate that this synthetic stimulus employed in this study robustly activates most auditory cortical areas bilaterally.

| ROI | Monkey M1 | | | | Monkey M2 | | | | Monkey M3 | | | |
| --- | --- | --- | --- | --- | --- | --- | --- | --- | --- | --- | --- | --- |
|  | Left hemi | | Right hemi | | Left hemi | | Right hemi | | Left hemi | | Right hemi | |
|  | β | p | β | p | β | p | β | p | β | p | β | p |
| A1 | 6.81 | 3e-5 | 3.47 | 0.04 | 4.15 | 1e-3 | 2.53 | 0.01 | 2.41 | n.s. | 2.73 | n.s. |
| AL | 3.63 | 0.03 | 6.52 | 3e-4 | 3.15 | n.s. | 1.58 | n.s. | 2.52 | n.s. | 3.02 | 0.01 |
| CL | 1.58 | n.s. | - | - | - | - | - | - | - | - | - | - |
| CM | 4.08 | n.s. | 5.22 | 4e-3 | 6.28 | 4e-5 | 3.95 | 0.01 | 1.82 | n.s. | 3.78 | n.s. |
| CPB | 0.97 | n.s. | 3.02 | n.s. | 0.81 | n.s. | -0.38 | n.s. | 1.52 | n.s. | 0.52 | n.s. |
| ML | 6.45 | 3e-3 | 1.38 | n.s. | -0.36 | n.s. | 0.77 | n.s. | 3.37 | n.s. | 0.56 | n.s. |
| R | 6.08 | 1e-6 | 6.71 | 1e-7 | 6.65 | 5e-6 | 3.72 | 1e-3 | 4.17 | n.s. | 3.81 | 0.02 |
| RM | -1.87 | n.s. | 3.03 | n.s. | 8.09 | 2e-6 | 4.42 | 0.05 | 3.46 | n.s. | 4.27 | 5e-3 |
| RPB | 0.12 | n.s. | 1.48 | n.s. | 1.18 | n.s. | 0.34 | n.s. | 1.05 | n.s. | 1.2 | n.s. |
| RT | -0.91 | n.s. | 0.44 | n.s. | - | - | - | - | 1.9 | n.s. | 3.38 | n.s. |
| RTL | -1.38 | n.s. | 0.09 | n.s. | 1.27 | n.s. | - | - | 1.03 | n.s. | 3.27 | n.s. |
| RTM | -1.81 | n.s. | 0.91 | n.s. | - | - | - | - | 3.39 | n.s. | 1.53 | n.s. |
| RTp | - | - | 1.32 | n.s. | - | - | - | - | 0.64 | n.s. | 2.5 | n.s. |
| STGr | - | - | 0.45 | n.s. | - | - | - | - | 1.07 | n.s. | 1.54 | n.s. |
| Tpt | -1.16 | n.s. | - | - | - | - | - | - | - | - | - | - |

Supplementary Table S2 Linear negative parametric contrast details from various fields in the auditory cortex of three monkeys.

Beta and significance value is given for ‘linear negative parametric’ contrast across all the ROIs in monkeys M1, M2, and M3. The beta is averaged across those voxels whose t-statistic for ‘sound versus silence’ meets p<0.001 uncorrected for multiple comparisons across the auditory cortex. In some ROIs, where the details are omitted, no voxels within this ROI survived this threshold at a single voxel level. The significance value (p-value) is corrected for multiple comparisons across ROIs within each monkey. (n.s. – not significant i.e. p > 0.05). This data indicate that BOLD decreases with increasing time window duration in most auditory core and belt cortical areas in these monkeys.

**Supplementary analysis - Slope of linear regression**

An alternate analysis to linear negative parametric contrast is to compute the slope of linear regression of the BOLD signal for different spectrotemporal correlation to the underlying time window duration. A positive slope indicates a preference for longer time window while a negative slope indicates a preference for shorter time window. This linear regression was performed using lm() function in R software. The fitted linear functions were of the form: $\beta\left( r_{1} \right)=m*w_{min}+c$, where $\beta$ is the regression coefficient averaged across those voxels within an ROI whose sound versus silence contrast is significant (T>3.1, p<0.001 uncorrected for multiple comparisons across the auditory cortex), $r_{1}$ is the spectrotemporal correlation, $w_{min}$ is the duration of time window showing a minimum correlation $r_{min}$, $m$ is the slope of the linear regression and $c$ is the y-intercept of the fit.

Supplementary Table S3 summarizes the slope of the linear regression against time window duration and its corresponding significance level from both hemispheres in monkey M1, M2, and M3 respectively across those ROIs where there were voxels whose sound versus silence contrast was significant (T>3.1, p<0.001 uncorrected for multiple comparisons across the auditory cortex). From the curve fit of BOLD contrast with time window duration using linear regression in each ROI where sound vs silence contrast was significant, we analysed the slope of the fitted line that conveys the degree of the relative preference towards different time window durations. Across three monkeys M1, M2, M3 (a total of 6 hemispheres), a negative slope was noticed in most auditory core and belt regions bilaterally and importantly positive slope was not statistically significant. This implied that most cortical areas prefer a shorter window over a longer window. The slope averaged across the hemispheres of three animals was as follows - in the core cortical regions (A1, R, RT): -4.77e-3; in the belt cortical regions (CM, RM, RTM, AL, ML, RTL, CL): -3.56e-3; in the parabelt cortical regions (CPB, RPB, RTp, STGr, Tpt): -1.41e-3; in the non-core cortical regions: -2.79e-3. Thus, the slope was steepest in the core cortical regions and this negative slope reduced, despite staying negative, as one moved to belt cortical regions (Welch Two Sample t-test core < belt, t(22.2) = -2.41, p < 0.012) which further reduced in parabelt cortical regions (Welch Two Sample t-test belt < parabelt, t(50.04) = -3.72, p < 2e-4; core < parabelt, t(15.26) = -5.46, p < 3e-5). This implied that the duration of the preferred window of temporal integration in a given cortical region widened as one progressed from core to belt cortical regions.

| ROI | Monkey M1 | | | | Monkey M2 | | | | Monkey M3 | | | |
| --- | --- | --- | --- | --- | --- | --- | --- | --- | --- | --- | --- | --- |
|  | Left hemi | | Right hemi | | Left hemi | | Right hemi | | Left hemi | | Right hemi | |
|  | m | p | m | p | m | p | m | p | m | p | m | p |
| A1 | -9e-3 | 0.03 | -5e-3 | n.s. | -6e-3 | n.s. | -3e-3 | n.s. | -2e-3 | n.s. | -4e-3 | 0.05 |
| AL | -5e-3 | 0.05 | -9e-3 | 0.04 | -4e-3 | n.s. | -1e-3 | n.s. | -2e-3 | n.s. | -4e-3 | 0.01 |
| CL | -3e-3 | n.s. | - | - | - | - | - | - | - | - | - | - |
| CM | -6e-3 | n.s. | -7e-3 | n.s. | -9e-3 | 0.01 | -5e-3 | 0.03 | -2e-3 | n.s. | -4e-3 | n.s. |
| CPB | -2e-3 | n.s. | -4e-3 | n.s. | -2e-3 | n.s. | 1e-3 | n.s. | -2e-3 | n.s. | -5e-4 | n.s. |
| ML | -9e-3 | 0.01 | -2e-3 | n.s. | -9e-4 | n.s. | -1e-3 | n.s. | -4e-3 | n.s. | -1e-3 | n.s. |
| R | -9e-3 | 0.05 | -9e-3 | 0.02 | -9e-3 | n.s. | -4e-3 | n.s. | -5e-3 | n.s. | -5e-3 | 0.01 |
| RM | 2e-3 | n.s. | -4e-3 | n.s. | -0.01 | n.s. | -4e-3 | n.s. | -5e-3 | 0.04 | -5e-3 | n.s. |
| RPB | -5e-4 | n.s. | -1e-3 | n.s. | -2e-3 | n.s. | -1e-3 | n.s. | -1e-3 | n.s. | -1e-3 | n.s. |
| RT | 6e-4 | n.s. | -2e-3 | n.s. | - | - | - | - | -4e-3 | n.s. | -4e-3 | 0.01 |
| RTL | 7e-4 | n.s. | -1e-3 | n.s. | -2e-3 | n.s. | - | - | -3e-3 | n.s. | -3e-3 | n.s. |
| RTM | 2e-3 | n.s. | -2e-3 | n.s. | - | - | - | - | -6e-3 | 0.05 | -3e-3 | n.s. |
| RTp | - | - | -3e-3 | n.s. | - | - | - | - | -3e-3 | n.s. | -3e-3 | n.s. |
| STGr | - | - | -4e-5 | n.s. | - | - | - | - | -2e-3 | n.s. | -2e-3 | n.s. |
| Tpt | 2e-3 | n.s. | - | - | - | - | - | - | - | - | - | - |

Supplementary Table S3: Slope of a straight line as a function of window duration fitted on beta from various fields in auditory cortex of three monkeys

. A straight line is fit on the betas as a function of window duration using linear regression and the slope of this line is determined along with its significance value. The beta used for each condition / correlation is averaged across those voxels whose t-statistic for ‘sound versus silence’ meets p<0.001 uncorrected for multiple comparisons across the auditory cortex. n.s. – not significant, p>0.05. A negative slope is noticed in most core and belt areas. Further, this slope reduces as one moves from core to non-core auditory cortical regions

## Supplementary References

Barton B, Venezia JH, Saberi K, Hickok G, Brewer AA. 2012. Orthogonal acoustic dimensions define auditory field maps in human cortex. Proceedings of the National Academy of Sciences of the United States of America. 109:20738-20743.

Baumann S, Joly O, Rees A, Petkov CI, Sun L, Thiele A, Griffiths TD. 2015. The topography of frequency and time representation in primate auditory cortices. Elife. 4.

Belin P, Zilbovicius M, Crozier S, Thivard L, Fontaine A, Masure MC, Samson Y. 1998. Lateralization of speech and auditory temporal processing. Journal of cognitive neuroscience. 10:536-540.

Bendor D, Wang X. 2007. Differential neural coding of acoustic flutter within primate auditory cortex. Nat Neurosci. 10:763-771.

Bendor D, Wang X. 2008. Neural response properties of primary, rostral, and rostrotemporal core fields in the auditory cortex of marmoset monkeys. J Neurophysiol. 100:888-906.

Boemio A, Fromm S, Braun A, Poeppel D. 2005. Hierarchical and asymmetric temporal sensitivity in human auditory cortices. Nat Neurosci. 8:389-395.

Brugge JF, Nourski KV, Oya H, Reale RA, Kawasaki H, Steinschneider M, Howard MA, 3rd. 2009. Coding of repetitive transients by auditory cortex on Heschl's gyrus. J Neurophysiol. 102:2358-2374.

Erb J, Armendariz M, De Martino F, Goebel R, Vanduffel W, Formisano E. 2019. Homology and Specificity of Natural Sound-Encoding in Human and Monkey Auditory Cortex. Cerebral cortex (New York, NY : 1991). 29:3636-3650.

Giraud AL, Lorenzi C, Ashburner J, Wable J, Johnsrude I, Frackowiak R, Kleinschmidt A. 2000. Representation of the temporal envelope of sounds in the human brain. J Neurophysiol. 84:1588-1598.

Herdener M, Esposito F, Scheffler K, Schneider P, Logothetis NK, Uludag K, Kayser C. 2013. Spatial representations of temporal and spectral sound cues in human auditory cortex. Cortex; a journal devoted to the study of the nervous system and behavior. 49:2822-2833.

Jamison HL, Watkins KE, Bishop DV, Matthews PM. 2006. Hemispheric specialization for processing auditory nonspeech stimuli. Cerebral cortex (New York, NY : 1991). 16:1266-1275.

Leaver AM, Rauschecker JP. 2016. Functional Topography of Human Auditory Cortex. The Journal of neuroscience : the official journal of the Society for Neuroscience. 36:1416-1428.

Liegeois-Chauvel C, Lorenzi C, Trebuchon A, Regis J, Chauvel P. 2004. Temporal envelope processing in the human left and right auditory cortices. Cerebral cortex (New York, NY : 1991). 14:731-740.

Lu T, Liang L, Wang X. 2001. Temporal and rate representations of time-varying signals in the auditory cortex of awake primates. Nat Neurosci. 4:1131-1138.

Niwa M, Johnson JS, O'Connor KN, Sutter ML. 2013. Differences between primary auditory cortex and auditory belt related to encoding and choice for AM sounds. The Journal of neuroscience : the official journal of the Society for Neuroscience. 33:8378-8395.

Nourski KV, Brugge JF, Reale RA, Kovach CK, Oya H, Kawasaki H, Jenison RL, Howard MA, 3rd. 2013. Coding of repetitive transients by auditory cortex on posterolateral superior temporal gyrus in humans: an intracranial electrophysiology study. J Neurophysiol. 109:1283-1295.

Overath T, McDermott JH, Zarate JM, Poeppel D. 2015. The cortical analysis of speech-specific temporal structure revealed by responses to sound quilts. Nat Neurosci. 18:903-911.

Overath T, Zhang Y, Sanes DH, Poeppel D. 2012. Sensitivity to temporal modulation rate and spectral bandwidth in the human auditory system: fMRI evidence. J Neurophysiol. 107:2042-2056.

Santoro R, Moerel M, De Martino F, Goebel R, Ugurbil K, Yacoub E, Formisano E. 2014. Encoding of natural sounds at multiple spectral and temporal resolutions in the human auditory cortex. PLoS Comput Biol. 10:e1003412.

Santoro R, Moerel M, De Martino F, Valente G, Ugurbil K, Yacoub E, Formisano E. 2017. Reconstructing the spectrotemporal modulations of real-life sounds from fMRI response patterns. Proceedings of the National Academy of Sciences of the United States of America. 114:4799-4804.

Schönwiesner M, Rübsamen R, Von Cramon DY. 2005. Hemispheric asymmetry for spectral and temporal processing in the human antero‐lateral auditory belt cortex. The European journal of neuroscience. 22:1521-1528.

Schonwiesner M, Zatorre RJ. 2009. Spectro-temporal modulation transfer function of single voxels in the human auditory cortex measured with high-resolution fMRI. Proceedings of the National Academy of Sciences of the United States of America. 106:14611-14616.

Scott BH, Malone BJ, Semple MN. 2011. Transformation of temporal processing across auditory cortex of awake macaques. J Neurophysiol. 105:712-730.

Steinschneider M, Reser DH, Fishman YI, Schroeder CE, Arezzo JC. 1998. Click train encoding in primary auditory cortex of the awake monkey: evidence for two mechanisms subserving pitch perception. J Acoust Soc Am. 104:2935-2955.

Zatorre RJ, Belin P. 2001. Spectral and temporal processing in human auditory cortex. Cerebral cortex (New York, NY : 1991). 11:946-953.
